# Recognition and diagnosis of sleep disorders in Parkinson’s disease

**DOI:** 10.1007/s00415-012-6505-7

**Published:** 2012-04-26

**Authors:** Maartje Louter, Willemijn C. C. A. Aarden, Joy Lion, Bastiaan R. Bloem, Sebastiaan Overeem

**Affiliations:** 1 Sleep Medicine Centre ‘Kempenhaeghe’, Heeze, The Netherlands; 2 Department of Neurology, Donders Institute for Brain, Cognition and Behaviour, Radboud University Nijmegen Medical Centre, PO Box 9101, 6500 HB Nijmegen, The Netherlands

**Keywords:** Parkinson’s disease, Sleep disorders, Excessive daytime sleepiness, Polysomnography

## Abstract

Sleep disturbances are among the most frequent and incapacitating non-motor symptoms of Parkinson’s disease (PD), and are increasingly recognized as an important determinant of impaired quality of life. Here we review several recent developments regarding the recognition and diagnosis of sleep disorders in PD. In addition, we provide a practical and easily applicable approach to the diagnostic process as a basis for tailored therapeutic interventions. This includes a stepwise scheme that guides the clinical interview and subsequent ancillary investigations. In this scheme, the various possible sleep disorders are arranged not in order of prevalence, but in a ‘differential diagnostic’ order. We also provide recommendations for the use of sleep registrations such as polysomnography. Furthermore, we point out when a sleep specialist could be consulted to provide additional diagnostic and therapeutic input. This structured approach facilitates early detection of sleep disturbances in PD, so treatment can be initiated promptly.

## Introduction

Sleep disorders are among the most common non-motor symptoms in Parkinson’s disease (PD), with an estimated prevalence of 65 % to more than 95 % [19, 41, 46, 63]. Sleep disorders negatively affect the quality of life [54, 55, 58]. Fortunately, specific treatment options are available, but adequate treatment requires a precise and timely diagnosis of the specific sleep disorder at hand. This recognition and diagnosis remain challenging in everyday clinical practice because of the wide variety and intricate combinations of sleep disorders in PD.

The whole gamut of sleep disorders may occur in PD, including excessive daytime sleepiness, insomnia, nocturnal motor symptoms and sleep-related breathing disorders. Three main groups of causes can be identified. Sleep problems can be a primary disease symptom caused by neuronal degeneration in sleep-regulating brain regions. An example is EDS in patients with cognitive decline [64]. Second, sleep may be disrupted by other symptoms of PD, such as nocturnal motor symptoms (e.g., difficulty turning in bed) or autonomic dysfunction (e.g., nocturia). Third, many drugs used in the treatment of PD can affect sleep [18, 29, 46, 48, 53]. For example, selegiline—which is metabolized to methamphetamine and amphetamine—may cause insomnia [12].

Most sleep disturbances can be diagnosed and treated by a movement disorders specialist. However, the diversity and complex origin of sleep disorders in PD may complicate the diagnostic trajectory. Ancillary investigations are needed occasionally, including polysomnographic recordings. In specific cases, the diagnostic and therapeutic help of a sleep medicine specialist can be useful.

To adequately treat sleep disorders in PD, an accurate diagnosis is crucial. Treatment options are diverse and depend on the specific sleep disorder(s) that are present. In Table 1, some of the most common sleep disorders are highlighted, together with specifically tailored treatment options. For more elaborate details on the treatment of sleep disorders in PD, we refer to previously published reviews [4, 44].

**Table 1:** Therapeutic options of the most common sleep disorders in PD

Excessive daytime sleepiness	Disrupted nocturnal sleep	Improve nocturnal sleep
	Medication side effect	If possible discontinue or change medication
	Primary hypersomnia	Stimulant medication • Modafinil [1] • Methylphenidate (no controlled studies)
Nocturnal motor symptoms	Nocturnal “off”	• Slow release levodopa preparation [37] • Continuous dopaminergic stimulation (apomorfine, rotigotine, duodopa) [56, 67]
	Restless legs syndrome, periodic limb movement disorder	• Increase nighttime dose of dopaminergic medication [25] • Opiate [25] • Gabapentin [25]
	REM sleep behavior disorder	• Clonazepam [59] • Melatonin [6]
Sleep-related breathing disorder	Obstructive sleep apnea	• Continuous positive airway pressure [44]
	Nocturnal stridor	• Continuous positive airway pressure [26, 35, 36] • Tracheotomy [61]

The purpose of this article is twofold. We highlight the most important recent developments that have clinical relevance for the (differential) diagnosis of sleep disorders in PD. In addition—and based on this new knowledge—we provide a practical, easily applicable approach to the recognition and diagnosis of sleep disorders in PD and atypical parkinsonian syndromes.

## Sleep diagnostics

### The sleep history

The clinical interview remains the single most important diagnostic instrument. Although sleep disorders are common in PD, they are not always mentioned spontaneously by the patient, as was recently shown [11]. A few quick screening questions, probing both nocturnal sleep and daytime sleepiness (see Table 2), should be asked on a regular basis in every PD patient. When these questions raise suspicion of a relevant sleep disorder, a structured history is the essential starting point of the diagnostic trajectory. Table 2 describes the various topics that should be covered in such a comprehensive sleep history.

**Table 2:** Key elements of the sleep history for PD patients

Screening questions
Sleep onset insomnia (sleep latency >30 min)
Frequent awakenings
Non-restorative sleep (unrefreshed in the morning, tiredness/sleepiness just after awakening)
Daytime sleepiness (either unwanted sleep episodes or napping)
When a sleep disorder is suspected
Check habitual bedtimes, sleep latency, number and duration of awakenings, total sleep time
Screening for nocturnal motor symptoms including ‘off’ symptoms and RBD
Screening for nocturia, nocturnal pain
Screening for sleep-related breathing disorders
Screening for mood and anxiety disorders, hallucinations
Daytime sleepiness: frequency, warning signs, driving
Detailed medication schedule, relation to sleep symptoms
Further questioning
Sleep relating breathing disorders
Snoring, witnessed apneas, nocturnal stridor, daytime stridor
Nocturia, night sweats, dry mouth in the morning, morning headaches
REM sleep behavior disorder
Sleep talking, shouting, swearing
Gross body movements resembling ‘dream enactment’ (often aggressive)
Restless legs syndrome
Check diagnostic criteria (see Table 3)
Nocturia
Frequency, volume, urologic symptoms during the day
Fluid intake in the evening, caffeine and alcohol use, medication such as diuretics
Primary insomnia
Circumstances around onset, sleep hygiene, extending bed times ‘to try and catch some sleep’
Worrying when lying awake, frequently checking the clock
Mood and other co-morbid disorders
Excessive daytime sleepiness
Frequency and duration of unintentional sleep episodes
Circumstances, warning signs, relation with dopaminergic medication
Driving, effect of planned naps

### Sleep questionnaires

Sleep questionnaires can help to collect data in a standard fashion, although they are no substitute for a personal clinical interview. In the past years, several sleep questionnaires have been developed to indentify sleep disorders in PD. A recent study of The Sleep Scale Task Force reviewed these scales and made recommendations for their use [31]. The Pittsburgh Sleep Quality Index (PSQI) is a well-validated measure of nocturnal sleep quality and severity of nighttime sleep disturbances [8]. The Parkinson’s Disease Sleep Scale (PDSS) is a more general scale that specifically rates sleep problems in PD [10]. The Epworth Sleepiness Scale (ESS) is recommended to screen for excessive daytime sleepiness and to rate its severity [38]. A specific screening for sleep attacks is provided by The Inappropriate Sleep Composite Score (ISCS) [30]. Nighttime sleep problems and excessive daytime sleepiness are both part of the Scales for Outcomes in Parkinson’s Disease Sleep (SCOPA-SLEEP), but this scale has not yet been validated against other—objective—sleep measures [43].

The Sleep Scale Task Force has also commented on the fact that many available sleep questionnaires offer an overall rating of the severity of night- or daytime sleep problems, but are not intended to diagnose a specific sleep disorder. Recently, the PDSS has been revised and updated to tackle this issue. The PDSS-2 now screens for sleep disorders that are common in PD, such as restless legs syndrome, nocturnal akinesia, and/or pain and sleep apnea [66]. The PDSS-2 was validated using a semi-structured interview, but a validation against objective measurements such as polysomnography has not yet been performed.

### Sleep registrations

A number of neurophysiological studies allow for the assessment of sleep architecture and the detection of nocturnal sleep disorders as well as excessive daytime sleepiness, but these sleep registrations should always be interpreted carefully and in combination with the clinical interview. The mainstay technique is polysomnography (PSG): the simultaneous recording of multiple signals to measure both sleep itself and associated physiological parameters such as breathing. Additional audiovisual recording can be very useful, especially for diagnosing nocturnal movement disorders, such as REM sleep behavior disorder. Excessive daytime sleepiness can be objectified using the Multiple Sleep Latency Test (MSLT). Poryazova et al. [52] found a significant correlation between ESS scores >10 (indicating excessive daytime sleepiness) and short mean sleep latency (≤5 min) as measured with the MSLT. Although in daily practice the clinical interview and additional questionnaires are often sufficient to diagnose excessive daytime sleepiness, an MSLT can be considered when an objective diagnosis is needed (e.g., in relation to driving) or when there is difficulty separating sleepiness from related complaints of fatigue.

### Diagnostic strategy

**Fig. 1:**
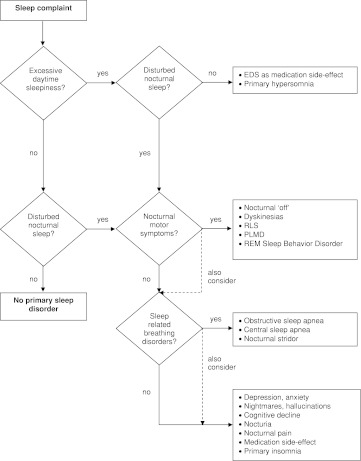
Diagnostic flowchart for the assessment of sleep disorders in PD

Most reviews on sleep disorders in PD are organized based on the (presumed) pathophysiology, but lack a clear structure that can aid in the differential diagnosis. Adopting a systematic way of thinking about disturbed sleep facilitates the clinical interview and reduces the risk of missing sleep disorders. In Fig. 1, we have put the various PD-related sleep disorders into a flowchart, which can be followed in every patient. The order is based on differential diagnostic grounds, and is not related to prevalence or severity. The various disorders are categorized into “excessive daytime sleepiness” and “disturbed nocturnal sleep.” The latter category is in turn subdivided into “nocturnal motor symptoms,” “sleep-related breathing disorders” and “other causes of insomnia.”

The diagnostic process starts with determining whether daytime sleepiness is present. If so, one should decide if disturbed nocturnal sleep is a likely culprit, in which case the flowchart should be followed in that direction. If not, side effects of medication and primary hypersomnias remain possible causes for excessive daytime sleepiness. Nocturnal motor symptoms are the primary cause to be checked when nighttime sleep is disturbed. Sleep-related breathing disorders should then be checked for. What remains are several other causes of insomnia, with their associated diagnostic trajectory. Importantly, one should always consider the fact that more than one sleep disorder can be present.

Table 3 explains the various categories of the flowchart, with the associated diagnostic tools. The table further shows in which cases the help of a sleep medicine specialist could be considered for additional diagnostic and therapeutic input.

**Table 3:** Diagnostic outline for sleep disorders in PD

	Main sleep symptom	Etiological category	Diagnostic strategy
Daytime	Excessive daytime sleepiness	Disturbed nocturnal sleep (see nighttime)	
		Medication side effect	Clinical history, dedicated questionnaires (e.g., ESS), lower dose or change to different drug
		**Primary hypersomnia**	**Clinical history (exclusion of other causes), dedicated questionnaires (e.g., ESS), MSLT**
Nighttime	Nocturnal motor symptoms	Nocturnal ‘off’: tremor, rigidity, akinesia, dystonia	Clinical history, when in doubt: video-polysomnography
		Dyskinesias	Clinical history
		Restless legs syndrome	Clinical criteria, laboratory investigations (e.g., ferritin levels)
		**Periodic limb movement disorder**	**Polysomnography (interpretation by sleep specialist)**
		**REM sleep behavior disorder**	**Clinical history, video-polysomnography**
	Sleep-related breathing disorders	**Sleep apnea syndromes (obstructive and/or central)**	**Clinical history, clinical examination, pulmonological evaluation, polygraphy/polysomnograpy**
		**Nocturnal stridor**	**Clinical history, polysomnography with synchronous audio recording, consider laryngoscopy**
	Other causes of insomnia	Depression, anxiety	Clinical history, diagnostic questionnaires, assessment by psychiatrist
		Nightmares, hallucinations, psychosis	Clinical history, assessment by psychiatrist
		Cognitive decline and dementia	Neuropsychological tests, check underlying treatable causes
		Nocturia	Clinical history, physical examination, urological evaluation
		Nocturnal pain	Clinical history, screen for comorbidity, physical examination
		Medication side effects	Clinical history, lowering dose or change to different drug
		**Primary insomnia**	**Clinical history, exclusion of other causes**

In bold typical indications for consultation with a sleep medicine specialist

*ESS* Epworth Sleepiness Scale, MSLT multiple sleep latency test

## Excessive daytime sleepiness

### Excessive daytime sleepiness versus ‘sleep attacks’

The most important characteristic of excessive daytime sleepiness (EDS) is the tendency to actually fall asleep during the day, usually accompanied by a ‘building’ feeling of sleepiness. Prevalence rates of EDS in PD range from 29 % to almost 60 % [27, 30, 48, 52, 64, 69]. The term ‘sleep attacks’ is often intermingled with EDS, but this is not justified. Sleep attacks are sudden, irresistible sleep episodes, often without warning signs. In the late 1990s, Frucht et al. [23] described sleep attacks in PD patients while driving after starting treatment with dopamine agonists. A large study from Canada, including 638 consecutive PD patients, showed a prevalence of sleep attacks while driving of 3.8 % [30]. In only 0.7 % of patients, sleep episodes occurred without any warning signs. Sleep attacks can also occur during activities other than driving. Paus et al. [51] reported sudden onset sleep while engaged in some form of activity (e.g., during a meal or phone call) in 580 of 2,952 patients (19.6 %). Körner et al. [39] suggested even higher prevalence rates, with sudden sleep episodes in 42.5 % of 6,620 PD patients, but only in 19.2 % during activities.

### Excessive daytime sleepiness versus fatigue

Although patients often describe EDS as ‘fatigue,’ these represent two distinct symptoms. Fatigue is a feeling of tiredness and lack of energy, but does not result in unwanted sleep episodes [21]. Moreover, EDS and fatigue are differentially associated with clinical features of PD [69]. Specifically, fatigue is associated with higher scores on the motor part of the Unified Parkinson’s Disease Rating Scale (UPDRS III), higher Hoehn and Yahr stage and higher scores on a depression rating scale. Conversely, EDS is associated with the disease duration and type of dopaminergic treatment; patients with non-ergot dopamine agonists had higher scores on the ESS [69].

### Causes of excessive daytime sleepiness

Many studies have described a correlation between EDS and the use of dopaminergic drugs [18, 29, 48]. A recent study reported a dose-dependent effect of mean overall levodopa-equivalent dose and EDS, as measured with both ESS and MSLT. Importantly, this study analyzed the use of dopamine agonists, either alone or in combination with levodopa, but not levodopa alone [52]. However, some authors found no relation between EDS and medication, and concluded that sleepiness was related directly to PD pathology [3]. Recent studies focused on the possibility that EDS in PD may be related to damage to the hypothalamic sleep-regulating hypocretin system. Thus, Fronczek et al. [22] found a significant decrease in the number of hypocretin neurons and a lower hypocretin-1 concentration in ventricular CSF in post-mortem material. In the same issue of Brain, Thannickal et al. [65] also described a decrease in hypocretin neurons, which correlated with disease stage. Unfortunately, these pathological findings do not translate into a clinically useful test: one study reported low hypocretin-1 levels in spinal CSF in only two out of eight PD patients with EDS, and other research groups found normal spinal CSF levels [5, 50, 52, 73].

## Nocturnal motor symptoms

Nocturnal motor symptoms are an important cause of sleep disturbances in PD, and include nocturnal ‘off’ symptoms and dyskinesias, in addition to primary sleep disorders such as restless legs syndrome (RLS), periodic limb movement disorder (PLMD) and REM sleep behavior disorder (RBD).

### Nocturnal “off”

Lees was the first to describe the phenomenon of nocturnal “off” in 1988, reporting that 65 % of PD patients had difficulty turning around in bed [41]. Nowadays it is widely appreciated that nocturnal “off” symptoms contribute to impaired mobility in bed, making it difficult to turn around or find a comfortable sleep position. Surprisingly, no one has studied the influence of impaired bed mobility on sleep quality and sleep structure. The diagnosis of sleep problems caused by nocturnal “off” is primarily based on the clinical interview. There is a major need for objective measurements to diagnose sleep disturbances caused by nocturnal “off” periods. Recently, Bossenbroek et al. [7] validated the use of a tri-axial accelerometer to measure the intensity of movements in addition to body position during sleep. This seems to be a promising new way to objectively measure nocturnal “off” periods in PD, even in the home situation.

### Dyskinesias

Levodopa-induced dyskinesias are sometimes more intense in the evening, for example because of a cumulative effect of repeated doses of long-acting dopaminergic agents. Biphasic dyskinesias can also be severe in the evening after patients have taken their final evening dose of medication. Patients may report that these dyskinesias hamper the onset of sleep or disturb sleep when dyskinesias return during nighttime arousals [47]. However, no formal data are available on the prevalence and influence of nocturnal dyskinesias on sleep in PD patients.

### Restless legs syndrome

The dopaminergic system plays an important role in the pathophysiology of RLS, so it is to be expected that the prevalence of RLS is increased in PD. However, this relation between RLS and PD is not completely clear. Epidemiological studies yielded conflicting results. Some authors reported increased RLS prevalence rates of up to 21 % in Caucasian PD patients [49]. In contrast, a recent study could not confirm this high prevalence, although the study population was on relatively high levodopa doses, which may have influenced the outcomes [70].

RLS and PD may share a therapeutic response to dopaminergic drugs, but other characteristics point to a differential or additional effect of PD pathology. Compared to idiopathic RLS patients, PD patients with RLS have a higher age of onset and less often a positive family history of RLS [49]. In addition, studies using transcranial sonography found a significantly decreased echogenicity of the substantia nigra in patients with idiopathic RLS compared to PD patients with or without RLS, and healthy controls [40, 57].

Regardless of the exact prevalence, it remains important to be vigilant for the presence of restless legs in PD, because RLS has a negative influence on quality of life, while the symptoms can often be treated satisfactorily. Note that specific PD symptoms—such as akathisia—may obscure the diagnosis of RLS. Therefore, it is important to adhere to the diagnostic guidelines, which emphasize the presence of all four core features (Table 4) [2]. Secondary RLS should be excluded using appropriate investigations. In the general population, iron deficiency is the most common cause of secondary RLS, and one study confirmed this association in PD patients with RLS [10]. Ferritin levels are the best marker for iron deficiency [2, 10].

**Table 4:** Diagnostic criteria for RLS^43^

*Required criteria*
• Uncomfortable and unpleasant sensations in the extremities (prickling, stinging, itching, ‘like crawling ants,’ sometimes described as pain), with an urge to move
• The sensations begin or worsen during inactivity
• The sensations and/or urge to move are partially or totally relieved by movement
• The sensations and/or urge to move display a circadian pattern: worse in the evening or night compared than the early morning; or only occurring in the evening or night
*Supportive features of RLS*
• Positive family history
• Clear beneficial response to dopaminergics
• Presence of periodic limb movements during sleep

### Periodic limb movement disorder

Periodic limb movement disorder (PLMD) often co-occurs with RLS, but is a distinct disorder. Literature on the presence of PLMD in PD is scarce. Arnulf et al. [3] found PLMD in 15 % of a PD patient cohort, which is clearly higher than population estimates (which range around 8 %) [60]. Another study found more periodic limb movements in patients with PD compared to controls, but also compared to patients with MSA [71]. It is often assumed that periodic limb movements result in arousals from sleep, resulting in daytime symptoms. However, Arnulf et al. [3] found no association between the presence of PLMD and daytime sleepiness. Indeed, periodic limb movements are often asymptomatic, making it difficult to assess their clinical significance [9].

### REM sleep behavior disorder

RBD is a parasomnia resulting from loss of normal atonia during REM sleep, leading to vigorous behavior with enactment of vivid and frightening dreams. The excessive motor activity may even result in injuries to the patient or bed partner [15]. During the last decade RBD has been shown to be a premarker of alpha synucleopathies such as PD [13, 33]. The prevalence of RBD in PD is estimated between 15 and 30 % [15, 24, 28].

Given the potential dangers and available treatment options, asking every PD patient and the bed partner for the presence of ‘dream enactment’ is recommended (see Table 1). Eisensehr et al. [17] found frequent misdiagnoses when RBD is established solely upon the clinical interview. As RBD also needs to be differentiated from other sleep disorders, such as obstructive sleep apnea, confusional arousals, nocturnal hallucinations and nocturnal frontal seizures, the threshold for doing a PSG should be low, even when the clinical interview is very typical [34].

The gold standard for diagnosis remains a PSG with simultaneous audiovisual recording. Video-PSG allows a confident diagnosis by showing an increase of tonic and phasic muscle activity in REM sleep, sometimes associated with actual motor behavior. The “SinBar group”—a collaboration between the sleep groups in Innsbruck and Barcelona—introduced an EMG montage protocol, and showed that simultaneous recording of the m. mentalis, m. flexor digitorum superficialis in the upper limbs and m. extensor digitorum brevis in the lower limbs provides the highest rate of phasic EMG activity during REM sleep in patients with RBD [20]. More recent work evaluated the diagnostic performance of this EMG montage, showing a sensitivity of 94.4 %, specificity of 47.2 % and negative predictive value of 41.9 % [32]. Until recently, however, no formal cutoff values were available to determine when phasic or tonic EMG activity in REM sleep is ‘too high.’ In 2010, Montplaisir et al. [45] established the first set of such parameters for idiopathic RBD, which can be used in a clinical or research setup. In addition to EMG-based polysomnographic methods, a polysomnographic video-based scale is now available to rate the severity of RBD [62].

In addition to its clinical relevance, RBD also sheds highly interesting new light onto the regulation of motor activity in PD. De Cock et al. [16] found a restoration of normal motor control during nighttime movements in association with RBD. The majority of bed partners of PD patients with RBD (87 %) observed an improvement in movement quality during RBD episodes (faster, stronger or smoother). Video-PSG studies indeed showed body movements during REM without obvious signs of Parkinsonism during REM. The exact meaning of these observations remains unclear, but it was hypothesized that these movements are somehow generated in the motor cortex, thus bypassing the defective extrapyramidal systems [16].

## Sleep-related breathing disorders

Sleep-related breathing disorders are relatively common among the general population. They are important to diagnose, as they may not only result in unrefreshing sleep and daytime sleepiness, but also negatively influence the long-term cardiovascular risk profile.

The most common forms of sleep-related breathing disorders are sleep apnea syndromes, divided into two types: obstructive sleep apnea (OSAS) and central sleep apnea. OSAS is characterized by repetitive episodes of complete or partial upper airway obstruction that occur during sleep. Two recent studies show no increased risk of OSAS in PD patients [14, 68]. In fact, one study even found a lower prevalence (27 % in PD, compared to 40 % in controls referred for daytime sleepiness) [14]. The frequency of sleep apnea did not differ between unselected PD patients and patients who were referred for sleepiness. Differences in the patient population as well as selection of controls may explain the different results across studies.

Established risk factors for OSAS—such as increased neck circumference and retrognathia—obviously also apply to PD patients. However, there is no correlation between the presence of OSAS and snoring, sleepiness or elevated body mass index in PD [68].

Although OSAS is not more prevalent in PD patients compared to controls, the prevalence rate is still 15–30 % [14, 68], so clinicians should remain vigilant for its presence. The diagnostic yield of PSG versus limited respiratory polygraphy to detect sleep apnea in PD has not been studied. Given the complexity of sleep disturbances in PD with a high frequency of combined disorders, we advocate the use of full PSG whenever a sleep registration is deemed necessary.

### Nocturnal stridor

Nocturnal stridor is a specific sleep-related breathing disorder, which is mainly associated with multiple system atrophy [42]. Its presence has therefore differential diagnostic value in the workup of an extrapyramidal disorder. In addition, stridor is associated with a decreased life-expectancy [72]. Stridor can be recognized clinically as an inspiratory vocalization with a strained, harsh and high-pitched (260–330 Hz) sound during sleep. To formally diagnose nocturnal stridor, a PSG with audiovisual monitoring should be performed.

## Other causes of insomnia

When nocturnal motor symptoms and sleep-related breathing disorders are excluded, several other causes of disturbed nighttime sleep remain. Together, these may jointly be referred to as ‘insomnia.’ Up to 60 % of PD patients complain about insomnia, especially sleep fragmentation and early morning awakenings [63]. Insomnia can take several different forms, manifesting as difficulty with initiating sleep, maintaining sleep, early-morning awakenings or a combination of these. However, the diagnosis ‘insomnia’ should be used with caution. ‘Insomnia’ can have widely varying causes, and the generic term does not discriminate between them. In Fig. 1, these causes of problems with sleep initiation or maintenance are listed.

## Conclusions

Sleep disorders are very common in PD, but still their recognition and diagnosis remain challenging. Our proposed diagnostic workup can serve as a basis for tailored therapeutic interventions. The diagnostic armamentarium is extended and refined all the time, for example yielding well-validated clinical questionnaires or new devices to measure body movements related to sleep in the home environment.
